# Peripheral Flicker Fusion at High Luminance: Beyond the Ferry–Porter Law

**DOI:** 10.3390/vision7010026

**Published:** 2023-03-20

**Authors:** Maydel Fernandez-Alonso, Will Innes, Jenny C. A. Read

**Affiliations:** 1 Biosciences Institute, Newcastle University, Newcastle Upon Tyne NE2 4HH, UKjenny.read@newcastle.ac.uk (J.C.A.R.); 2 Translational Sensory and Circadian Neuroscience, Max Planck Institute for Biological Cybernetics, 72076 Tübingen, Germany; 3 Newcastle Eye Centre, Royal Victoria Infirmary, Newcastle Upon Tyne NE1 4LP, UK

**Keywords:** flicker fusion, critical flicker frequency, Ferry–Porter law, temporal perception, psychophysics

## Abstract

The relationship between luminous intensity and the maximum frequency of flicker that can be detected defines the limits of the temporal-resolving ability of the human visual system, and characterizing it has important theoretical and practical applications; particularly for determining the optimal refresh rate for visual displays that would avoid the visibility of flicker and other temporal artifacts. Previous research has shown that this relationship is best described by the Ferry–Porter law, which states that critical flicker fusion (CFF) increases as a linear function of log retinal illuminance. The existing experimental data showed that this law holds for a wide range of stimuli and up to 10,000 Trolands; however, beyond this, it was not clear if the CFF continued to increase linearly or if the function saturated. Our aim was to extend the experimental data available to higher light intensities than previously reported in the literature. For this, we measured the peripheral CFF at a range of illuminances over six orders of magnitude. Our results showed that for up to 10^4^ Trolands, the data conformed to the Ferry–Porter law with a similar slope, as previously established for this eccentricity; however, at higher intensities, the CFF function flattens and saturates at ~90 Hz for a target size of 5.7 degrees, and at ~100 Hz for a target of 10 degrees of angular size. These experimental results could prove valuable for the design of brighter visual displays and illumination sources that are temporally modulated.

## 1. Introduction

The interest in studying the temporal sensitivity of the human visual system has been closely related to the development of new visual display technologies. The introduction of the cinema at the end of the nineteenth century stimulated an early set of studies, while the beginning of widespread television use created another big push from the 1950s onwards. The reason was that these display technologies could present a bothersome flicker artifact caused by the successive presentation of the different frames. The critical flicker fusion (CFF) is the lowest frequency at which an intermittent light appears to be completely steady to the average human observer. The CFF is a very important concept for nearly all display technologies, determining the refresh rate at which they operate.

One early observation was that the frequency up to which flicker could be observed increased linearly with the logarithm of the luminance. This was observed by Ferry in 1892 and Porter in 1902 (cited in [1]), and it is known as the Ferry–Porter law. While other laws have existed that attempt to describe this relationship, Tyler and Hamer [2] demonstrated in a seminal study that the CFF follows this law at photopic levels and up to log_10_ 4 Trolands of retinal illuminance. This linear relationship is maintained even when the spectral composition, size and eccentricity of the stimulus change [1,2,3], although the slope and intercept of the function will vary depending on these factors.

Changes in the spectral composition of the test stimulus will alter the slope of the linear function between log illuminance and CFF. When the CFF is measured for green (510–555 nm) and red flicker (630–660 nm) as a function of retinal illuminance in both the fovea and peripheral retinal areas, the slope of the linear function is significantly steeper for the green stimuli [3]. The slope also varies as a function of the eccentricity of the target in the visual field. For stimuli of equal illuminance and size (scaled to match the anatomical density of receptors in different retinal areas), the slope of the CFF as a function of illuminance will become steeper as eccentricity increases by up to 40° temporally [4], and from there it will stay constant or decrease in some meridians. Finally, for a wide range of photopic luminance levels, the CFF increases linearly with the logarithm of the stimulus area, which is known as the Granit–Harper law [5]. Variations in the slope of the function reflect changes in the speed of the temporal response, while changes in the intercept reflect differences in the absolute threshold sensitivity. A stimulus of a larger size would stimulate a higher number of cells in the retina, allowing for more light to be captured, and thus increasing the absolute sensitivity to light. On the other hand, the steeper slope and decrease in the time constant in the periphery of the retina has been postulated to be due to an increase in the diameter of the cones’ inner and outer segments with eccentricity, which correlates with the generation of higher voltages in the phototransduction process, producing a proportional increase in sensitivity with increasing light intensity [6]. Finally, Hamer et al. [3] hypothesized that the increase in slope for green when compared to red light reflects a faster temporal processing for transmitting information near the CFF in the M cone pathway, with flicker detection being determined by the receptor mechanism that is most sensitive to the wavelength composition of the stimuli.

Several models exist that can predict how the CFF and log illuminance function can vary depending on the size and eccentricity of the target [7,8], with most predicting that the Ferry–Porter law continues to hold as intensity levels increase logarithmically. However, no experimental data exist of peripheral flicker sensitivity at intensities higher than log_10_ 4 Trolands. If, as predicted by these models, the CFF continues to rise linearly with log illuminance, this would mean that brighter displays would need very high refresh rates to avoid the perception of flicker, and even higher to reduce the visibility of other temporal artifacts. If, on the contrary, the response saturates at higher intensities, there would be no need for increasingly higher refresh rates.

Currently, several manufacturers have consumer-ready television displays that can reach peak luminance values between 2000 and 5000 cd/m^2^ [9]. This would correspond with retinal illuminance values between log_10_ 4.1 and log_10_ 4.5 Trolands—assuming a pupil diameter of 3 mm and after correcting for the Stiles–Crawford effect [8]. However, prototypes already exist of television screens that can reach up to 10,000 cd/m^2^ [10,11], which would amount to log_10_ 4.8 Trolands for a 3 mm pupil, and even novel micro-displays with intended applications in virtual and augmented reality, which can reach up to 3 million cd/m^2^ [12] or log_10_ 7.3 Trolands, exist. Although these high-luminance digital displays are not yet widely used or available to the public, mostly due to cost limitations, we can expect that, as these technologies continue to be developed, they will become more commonplace. This raises the need for extending the experimental data available on the human CFF at high-illuminance values. Furthermore, these results would also be relevant for illumination sources that are temporally modulated, such as LEDs, which are frequently controlled through pulse-width modulation. 

Some studies, which preceded the work of Tyler and colleagues, had found a saturation in the log illuminance-CFF function. Notably, Hecht et al. [13] found that in the fovea, the linear relationship held only up to approximately log_10_ 2 Trolands, at which point it saturated and started to decrease. The maximum frequency of flicker detected before saturation reached was between 50 to 60 Hz. Their results in other retinal eccentricities tested were similar, albeit paradoxically, they found that the slope was shallower in the periphery, and saturation was reached at lower illuminances. Indeed, most experimental results preceding the study by Tyler [4] had shown a slower response and lower sensitivity to flicker in the periphery [7,13,14,15]. However, Tyler at al. [2] later showed that with more careful control of the experimental setup and adjusting the size of the target to stimulate a similar number of photoreceptors at different retinal locations, the temporal response was indeed faster and sensitivity to flicker was higher in the periphery.

In summary, the relationship between the luminous intensity and the maximum frequency of flicker that can be detected defines the limits of the temporal-resolving ability of the human visual system, and characterizing this relationship has very important theoretical and practical applications. The CFF has been demonstrated to follow the Ferry–Porter law and reach up to approximately 90 Hz at 10,000 Trolands [2]; however, not much is currently known about the human CFF beyond these retinal illuminance levels. In this study, we aim to extend these measurements in the periphery at higher intensity levels than previously reported in the literature, and test whether the CFF continues to increase linearly with log intensity or if saturation is reached.

## 2. Materials and Methods

### 2.1. Participants

Data were collected from 5 adults aged between 25 and 45 years (mean 30.6, SD 8.26), out of which 2 were female and 3 were male. Due to the time requirements involved in the experiment, participants were recruited from postgraduate students and staff of the Institute of Biosciences at Newcastle University. The study was approved by the Newcastle University’s ethics committee (reference number 445487/2018) and written consent was obtained from each subject at the beginning of the first session.

### 2.2. Apparatus

**Figure 1:**
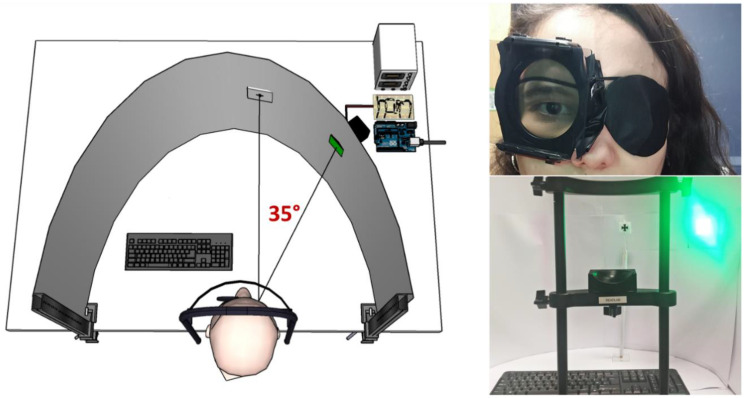
Graphical representation and photos of the experimental setup.

The experimental setup and apparatus were built following the one described in Tyler and Hamer (1990). A graphical representation and photos are provided in Figure 1.

The stimulus consisted of six high-power LEDs placed behind a diffuser with an angular size of 5.7 degrees. In the second task, this size was increased to 10 degrees. The diffuser was placed at 35 degrees of eccentricity from the right eye in the horizontal meridian, a retinal region chosen due to having a good homogeneity of receptors and peak temporal response [2]. The target LEDs had a peak wavelength of 526 nm and were relatively narrowband (FWHM: 24 nm), which guaranteed maximum luminous efficiency, while avoiding any risks associated with high intensity short wavelength light. The visual field was surrounded by a high luminance white background, which was used to keep a constant light adaptation level throughout the retina. A fixation cross was placed immediately in front of the right eye to help participants keep the test stimulus in the right location.

To create different levels of retinal illuminance without interfering with the stimulus, neutral density (ND) filters were used. These were placed over the observer’s eye during the experiment. Luminance measures of the stimulus were taken with a Konica Minolta LS-100 through all the filters used and with no filter. To maximise retinal illuminance during the experiment, the participant’s right pupil was dilated using eye drops of Cyclopentolate Hydrochloride at 1%, which also allowed to keep a constant pupil size throughout the experiment. 

A computer running MATLAB [16] controlled the experimental routine, selecting the stimulus levels and collecting and processing the participant’s responses. The frequency of the flicker was set via an Arduino Uno connected to the computer, which modulated the voltage of the LED driver circuit in a 50% duty cycle square wave.

**Figure 2:**
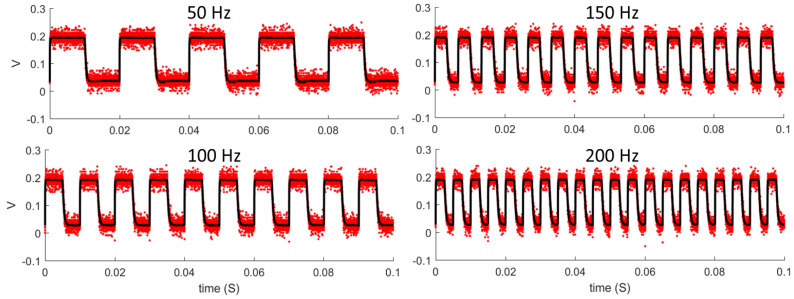
Luminous output measurements at four different frequencies. The red dots represent the raw individual measurements, while the black markers represent the average of these measurements at each time point.

To modulate the high-power LEDs at the required frequencies with minimal wave distortion, a fast-switching driver circuit was designed and built. High power LEDs can have large junction capacitance, which, added to the parasitic capacitance of the support circuitry, can slow the transitions and increase the rise and fall times of the luminous output. This would result in a non-square luminance waveform and would cause the total luminance to vary when the half-period of the flicker was less than the rise time of the LED. To address these issues, a custom circuit was designed and built, which minimized the changes in voltage between the ON and OFF states of the LEDs. Rather than switching the voltage to zero in the OFF state, a voltage was selected that was as close as possible to the ON state voltage, while generating the lowest possible luminous output. This output was sufficiently low to be absorbed by the box and diffuser where the LEDs were encased, and several tests were carried out to confirm that the luminance in the OFF state was 0 cd/m^2^. This reduction in voltage changes reduced the rise and fall time of the LEDs significantly, allowing the square waveform to be preserved (see Figure 2) and the total luminance to remain constant regardless of the frequency. 

Several calibration procedures were carried out on the experimental setup. Firstly, to confirm that the frequency of the flicker of the luminous output was equal to the input frequency, luminance measurements were taken with a photodiode connected to an oscilloscope (PicoScope 2000). All frequencies between 20 and 200 Hz, in steps of 10 Hz, were tested for 100 ms with 32 repetitions at each frequency. Some examples of these measurements are shown in Figure 2. All repetitions were averaged, and the resulting period was obtained and fitted in a linear regression against the input period. The resulting slope was equal to 1 and the intercept was 0.37 ms; thus, indicating a very good agreement between input and output. 

Secondly, to confirm that the luminance did not vary with the frequency of the flicker, measurements were taken with a Konica Minolta LS-100 at different frequencies between 40 Hz and 1000 Hz. No change in luminance with frequency was found, and the standard deviation of the measurements was well within the instrument error. Thirdly, to assess if luminance changed over time, the luminance measurements of the stimulus were taken over the course of 9 h, simulating the scenarios where multiple participants would perform the experiment in one day. We found that the luminance decreased by approximately 1300 cd/m^2^ over the first 5 min, beyond which no further consistent changes were found, with only small random fluctuations around the mean of 23,250 cd/m^2^ with a standard deviation of 204 cd/m^2^. To account for the small decrease at the start, we ensured the experimental setup was turned on for at least 15 min before commencing the data collection. 

### 2.3. Task and Design

Flicker fusion thresholds were measured by a YES/NO task using the constant stimuli method. One threshold estimate was obtained for each retinal illuminance level in each experimental session. To obtain the estimate, 8 equally spaced frequencies were presented 30 times each, in addition to 30 supra-threshold frequency trials (500 Hz) or “no flicker” trials, all in a randomized order. Between trials, the frequency was set to 1000 Hz. Eight levels of retinal illuminance were evaluated for each participant, from 3 to 6.5 log Trolands, approximately (actual values varied for each participant depending on their dilated pupil size).

A more advanced adaptive staircase procedure for YES/NO tasks was initially trialled: the quick yes–no algorithm [17]; however, after extensive testing, it was deemed to be unsuitable for our experiment, due to an overestimation of the threshold by approximately 15 Hz (±7 Hz) when compared to the more robust constant stimuli method. Further details can be found in [18].

### 2.4. Procedure

Before the day of the experiment, potential participants received the information sheet with all the details of the experiment, including the possible side effects of the drug used. On the day of the experiment, participants were given the information sheet to read, a consent form to sign, and an additional information leaflet to take with them, which included all the prevention measures, possible side effects of the drug and the steps to follow in case of an emergency. Once consent was obtained, their right pupil was dilated with two drops of Cyclopentolate Hydrochloride at 1%, and after a period of one hour, pupil diameter measurements were taken using the PowerRef 3, an infrared autorefractor with pupillometry capabilities. 

Participants then sat in front of the experimental setup with their head fixed in a chinrest. They viewed the stimuli through their right eye, which had the mask holding the corresponding ND filter, while the left eye was occluded with an eye patch. A cross central to the right eye was used as a fixation point. Instructions for the task were given, as well as a few trials of practice in their first session. In each trial, the stimulus was presented for an unlimited duration, until participants gave a response. Each experimental session had an approximate duration of 3 h, and frequent breaks were given between experimental blocks (every 20 min approximately). Participants completed between 3 and 8 sessions on separate days.

### 2.5. Threshold Estimation

The CFF thresholds were obtained following the procedure described in [19] for YES/NO tasks, and using the Palamedes toolbox [20] and custom MATLAB code.

In a YES/NO task, the observer must report in each trial whether the signal of interest (here, the flicker) is present or absent. The response given will be a function of the sensitivity of the observer to the specific stimulus presented (here, the CFF threshold representing the sensitivity to flicker), but also, of the inclination of the observer to judge one way or the other. This is termed the observer’s response bias, and can vary between different subjects, but also within the same observer across different sessions. Thus, it is important to find a way to measure the CFF thresholds independently of observer bias. The signal detection theory (SDT) offers a framework for obtaining bias-free estimates of perceptual thresholds.

One of the fundamental ideas of SDT is that neural responses are intrinsically noisy. This means that a specific frequency of flicker will not generate the same exact neural representation every time it is presented. Instead, the internal representation of the signal will vary randomly from trial to trial, taking the form of a normal probability distribution, with a mean that corresponds to the intensity of the signal (i.e., the frequency of flicker). SDT also postulates that neural noise is always present, so even in the absence of a stimulus, internal representations will be generated, which will vary randomly, also taking the form of a normal distribution. The extent to which the distribution of a signal overlaps with the noise distribution will determine the sensitivity to that signal. Indeed, a commonly used measure of sensitivity is *d*′ (d prime), which is the difference between the means of the signal and the noise distributions, in units of the standard deviation.

Due to the overlap between the noise and the signal distributions, observers must adopt a criterion of how strong the internal representation must be before they give an affirmative answer. Whenever the internal representation falls above this criterion, observers will indicate that the signal is present. The criterion adopted can be anywhere from rather strict (only a very strong internal response will elicit an affirmative answer), to rather loose (even a faint signal will result in an affirmative answer). A looser criterion will result in the observer having many hits (correctly identifying that flicker is present), but also many false alarms (indicating that flicker is present when it is actually absent). Conversely, a stricter criterion will result in fewer false alarms, but also in fewer hits. Since the criterion adopted can vary between observers and within the same observer across sessions, as mentioned previously, it is important to take it into account in order to estimate the CFF thresholds.

According to standard SDT, we can calculate the observer criterion (*C*) from the proportion of false alarms and the proportion of hits for each frequency of flicker, such that

$$C=-\frac{z\left(pH\right)+z\left(pF\right)}{2}\;$$

where *z(pH)* and *z(pF)* are the z-values for the proportion of hits and false alarms, respectively. Negative values of *C* indicate a bias toward “yes” responses (loose criterion), and positive values a bias toward “no” responses (strict criterion). *C* = 0 is the neutral criterion, with no bias in either direction.

Furthermore, it is also possible to estimate the sensitivity (*d*′) of the observer to each frequency of flicker by obtaining the difference between the means of the noise and the signal distributions, normalized to their standard deviation:

(1) $$d^{\prime }=z\left(pH\right)-z\left(pF\right)$$

One problem encountered on some occasions was that *d*′ would assume negative values whenever the proportion of hits for a specific frequency was lower than the proportion of false alarms. This would happen particularly in the higher frequencies (or lower periods of flicker) that were not visible to the observer. This is unlikely to be caused by a higher sensitivity to the null stimulus than to the frequency of the flicker presented, and more likely by random fluctuation about a small or zero *d*′, combined with relatively low number of trials at each level. Thus, whenever *d*′ assumed negative values, it was set to zero instead.

Finally, from the calculated *d*′, we obtained an unbiased measure of performance, that is, the percentage of correct responses the observer would achieve if a neutral criterion (C = 0) were adopted. This percentage, termed *Pc_ma_*_x_, is calculated as

(2) Pcmax=Φd′2

Once *Pc_max_* as a function of the period of flicker was obtained, it was fitted with a Quick psychometric function [19] through a maximum likelihood procedure, to obtain the estimates of the threshold, slope and lapse rate (which were allowed to vary, but constrained between 0 and 0.03). Standard errors of the threshold estimates were obtained through parametric bootstrap analysis with 600 simulations. Goodness-of-fit measurements based on the likelihood ratio test were then obtained for all psychometric function fits, and those that were deemed to be poor (*p* < 0.05) were discarded. This resulted in just one threshold estimate among all subjects being excluded.

**Figure 3:**
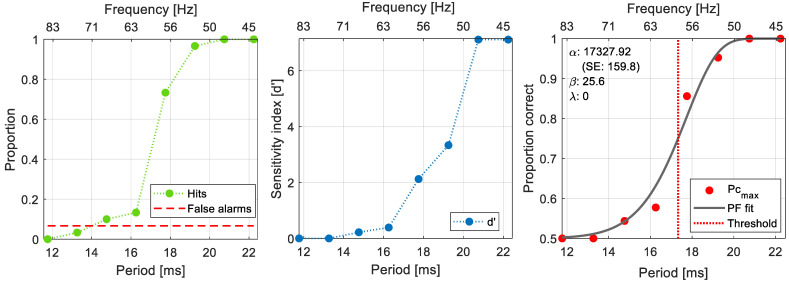
Example of the results obtained for one participant at one retinal illuminance level. The left panel shows the proportion of hits, as a function of the period of flicker, and the proportion of false alarms. The middle panel shows the calculated *d*′ as a function of the period of flicker presented. Note that when the proportion of hits is lower than the proportion of false alarms, *d*′ is set to 0. The right panel shows the unbiased proportion correct (*PC*_*max*_) as a function of the period of flicker, the Quick psychometric function fitted, and the estimated threshold. In all panels the corresponding frequency of flicker is displayed in the top abscissa.

In Figure 3, we provide an example of the calculation of *d*′ from the proportion of hits and false alarms, as well as the subsequent calculation of *Pc_max_* and psychometric function fit.

### 2.6. Data Analysis

Once the CFF thresholds were estimated, retinal illuminance was calculated from the luminance and the dilated pupil diameter, while correcting for the Stiles–Crawford effect, using the method described in [8], such that

(3) I=πd24L1−d9.72+d12.44

where *I* is retinal illuminance in Trolands, *L* is luminance in cd/m^2^ and *d* is the pupil diameter in mm.

The CFF thresholds as a function of log retinal illuminance for each participant were fitted with a two-segment piece-wise linear regression using the Segmented R library [21,22]. These data were further analysed by fitting a linear mixed model using MATLAB, with predictors of log retinal illuminance and squared log retinal illuminance. For the data obtained with the 5.7° target size, random effects of participant in these two predictors and the intercept were included, as well as random effects of the different experimental sessions within participant on the intercept. For the 10° target size, due to the smaller number of subjects and sessions, only a random effect of experimental session within participant on the intercept was included. The residuals of the models were inspected with diagnostic plots to confirm that no assumptions were violated.

## 3. Results

The data presented here and the processed results are available in the Newcastle University research repository with the identifier https://doi.org/10.25405/data.ncl.21725336 (accessed on 13 December 2022) [23]. The code used for the analyses and to generate the figures are also available in this repository with the identifier https://doi.org/10.25405/data.ncl.21764885 (accessed on 13 December 2022) [24].

In Figure 3, we present an example of the results obtained from one participant at one intensity level. As shown, for each experimental session and at each retinal illuminance value tested, we obtained the proportion of hits as a function of the period of flicker (green dots, left panel), and the proportion of false alarms (red dashed line). From these, we calculated *d*′ (middle panel), and from there the proportion of correct responses that would have been observed if the observer had been unbiased. This was fitted with a psychometric function to obtain a threshold estimate (right panel).

**Figure 4:**
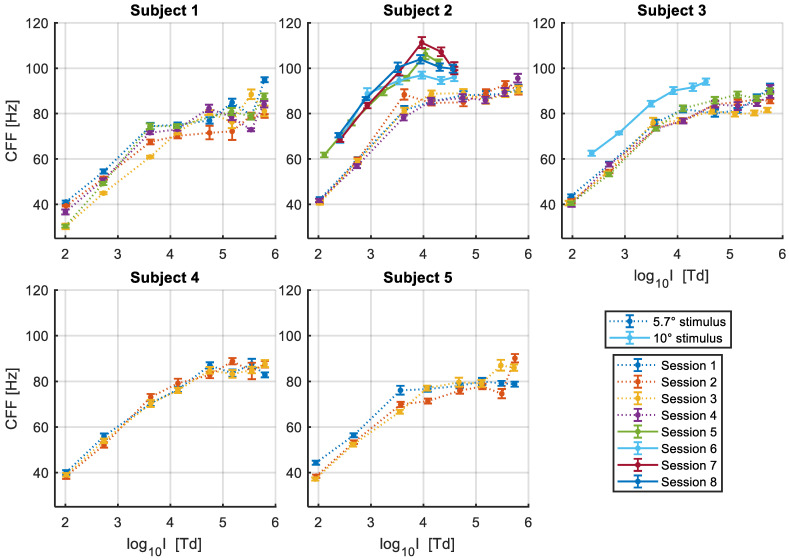
Estimated CFFs as a function of log_10_ retinal illuminance. Error bars represent the standard error of the CFFs obtained through parametric bootstrap analysis. Different sessions are represented with different colors. The dotted lines represent CFFs obtained for a stimulus of 5.7 degrees of visual angle, and the continuous lines for a stimulus of 10 degrees of visual angle.

The thresholds obtained as a function of log_10_ retinal illuminance (log I) for each participant in each experimental session are presented in Figure 4. For all subjects, the CFFs were measured for a test field size of 5.7 degrees of visual angle, while with two of the subjects we also collected data for a stimulus diameter of 10 degrees of visual angle. In general, we see that the CFF thresholds rise linearly with log I up to approximately log_10_ 4 Trolands, at which point it saturates between 80 and 90 Hz for the 5.7° stimulus. The larger stimulus diameter of 10°, placed at the same central eccentricity, increased the intercept of the function, and accordingly, the value at which it saturates (~100 to 110 Hz), but shows a similar slope. This means that the absolute sensitivity to flicker increases, but the rate at which it changes with increasing intensity is the same. We can also see that the point at which the response starts saturating is similar as for the smaller stimulus size within the same subject. Overall, there is considerable inter-subject variability in both the slope of the linear portion and the value at which the function asymptotes, as well as intra-subject variability between sessions for some participants. 

Some outlier CFF estimates at the highest intensity values can be observed for a few subjects (e.g., Subjects 1 and 5). Given the CFF estimates obtained at lower illuminance values, as well as the estimates obtained at that level of intensity in other experimental sessions, it is unlikely that these reflect a real detection of flicker and are more likely to be caused by noise in the psychophysical measurements and by perceptual artifacts that become more salient at the highest intensity levels.

### 3.1. Segmented Linear Regression

The apparent shape of the log illuminance—CFF function seems to vary between subjects. For some (e.g., Subjects 2, 3 and 4) the data could be well-described by a sigmoid function, with the rate of change on CFF with log illuminance progressively decreasing at higher intensities until it reaches saturation. For other subjects (e.g., Subjects 1 and 5) there seems to be a more sudden transition in the response. Since previous experimental data in the literature have shown this relationship to be linear up to log_10_ 4 Trolands, and to facilitate the comparison of results, we chose to firstly fit the data with a piece-wise linear function with two segments. For this, we used the Segmented R library, which allows us to estimate the breakpoint and its confidence intervals directly from the data without making any initial assumptions [21,22]. The results for each individual participant and the average of the parameter estimates are shown in Table 1.

We see that the breakpoint (i.e., the point at which the rate of change in the response changes abruptly) had similar estimates in most participants and in the two stimulus sizes used, with estimated values between 3.61 and 3.87 log_10_ Trolands, an average of 3.82 for the 5.7° target (mean 95% CI from 3.45 to 4.20 log_10_ Trolands) and 3.80 for the 10° target (mean 95% CI from 3.47 to 4.13 log_10_ Trolands). This suggests that the CFF to log illuminance function starts to saturate at an approximately similar value regardless of target size. For one subject, however, the estimated breakpoint is much higher at 4.45 log10 Trolands (95% CI from 4.22 to 4.68 log10 Trolands). This can be observed in Figure 4, where the CFF for this participant continues to increase relatively linearly at intensities where the responses of other observers have already saturated.

The slope of the first linear segment (i.e., the response at intensities lower than the breakpoint) shows further individual differences between observers, ranging from 18.2 Hz/decade to 25.5 Hz/decade, with an average of 21.0 Hz/decade for the 5.7° target size (mean 95% CI from 17.4 to 24.6 Hz/decade) and 21.7 Hz/decade for the 10° target size (mean 95% CI from 17.3 to 26.1 Hz/decade). Furthermore, we see that the estimated slope is consistent within participants across target sizes, with Subjects 2 and 3 having similar estimated rates of increase with overlapping confidence intervals in both conditions. These results indicate that, as expected, the size of the target does not affect the slope of the CFF—log illuminance function. 

Instead of the estimated y-intercept of the first linear segment, we report here the x-intercept instead, which was calculated from the estimated slopes and y-intercepts. The x-intercept is a more physiologically relevant measurement, as it represents the threshold sensitivity to light under given experimental conditions. We see that the estimated x-intercept is, as expected, much lower for the 10° target size with estimates between −0.82 and −0.54 log_10_ Trolands, when compared to the 5.7° target size (with estimates ranging from −0.19 to 0.42 log_10_ Trolands), reflecting the increased sensitivity to the same amount of light per unit area of the retina, when more photoreceptors are stimulated. 

Finally, there is large variability in the estimated slopes for the second segment of the piece-wise linear regression, with estimates ranging between 1.1 and 6.3 Hz/decade for the 5.7° target, and between −9.2 and 6.2 Hz/decade for the 10° target. The fact that most estimated values are positive might be reflecting the fact that the change in the response is not completely abrupt but rather gradual for most subjects, as well as the existence of some outliers at higher intensities. However, it is unlikely that the CFF would continue to rise at the estimated rates, as the response seems to completely saturate at the highest retinal illuminance values. Thus, to better capture both the gradual nature of the change and the saturation of the response, we performed a further analysis fitting the data with a quadratic function, using the more robust linear mixed model method. This approach will also allow us to better capture the between-subject and within-subject (i.e., between-session) variability in the measured CFF thresholds.

### 3.2. Linear Mixed Models

To better capture the relationship between CFF and log illuminance, we fitted a linear mixed-effects model to the thresholds obtained for each target size, with fixed effects of log illuminance and squared log illuminance. For the 5.7° target size, the intercept was allowed to vary randomly among subjects, with an additional random effect on the intercept of experimental session nested by subjects. More complex random effects structures that included the effects of log illuminance and squared log illuminance were tried but including these resulted in overfitted models, indicating that these structures were too complex to be supported by the data. For the CFF thresholds obtained with a 10° target size, we fitted a mixed model with the same fixed effects, but only a random effect of session nested within subject on the intercept. Thus, the intercept of the function could vary randomly for each experimental session completed by each participant, but not the other parameters of the model. Including a random effect of subject would not be advisable with only two subjects in this experimental condition, and allowing the effect of the other parameters to vary randomly by experimental session would add unsupported complexity to our model. 

**Figure 5:**
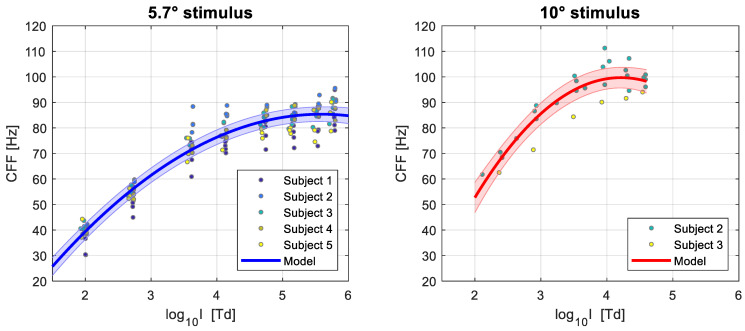
Quadratic linear mixed model results of CFF as a function of log_10_ retinal illuminance, for stimulus sizes of 5.7 (**left**) and 10 (**right**) degrees of visual angle. The continuous colored lines represent the model fit and the shaded regions represent the 95% confidence intervals. The individual markers show the estimated thresholds for each subject over several experimental sessions, and the color of the markers represents the participant as indicated by the legend.

The results of the fitted models are shown in Table 2 and illustrated in Figure 5. For the 5.7° target we see that the more gradual nature of the change in the CFF—log illuminance relationship has been more accurately captured, with the response completely saturating at the highest intensity values and for frequencies of flicker below 90 Hz. Note that the estimates of the model refer to the “average observer” given our sample, but as can be seen, some subjects can perceive flicker at higher frequencies than this. Using the random effects of the model (i.e., the parameters obtained for individual observers) would allow us to obtain estimates of the CFF at different light intensities for these more sensitive individuals. 

Somewhat similar results are seen for the 10° target, albeit with higher CFF thresholds for equal values of intensity, as previously discussed. The function seems to saturate at approximately 100 Hz and at similar values of light intensity as with the 5.7° stimulus. Overall, however, the limited number of subjects and data in this experimental condition result in a poorer fit with larger standard errors, and thus, greater uncertainty about the parameter estimates.

Finally, one more factor to consider is that the way the CFF thresholds were estimated here, and are usually estimated in the literature, might not be ideal when considering practical applications such as minimising the visibility of flicker on digital displays or illumination sources. As it is common in psychophysics, the CFFs reported here corresponded to the frequency at which subjects reported seeing flicker in 50% of the trials of the YES/NO task (equivalent to 75% of correct responses when considering the trials where no flicker was presented). However, in a real-life scenario, a temporally modulated illumination source that leads to the percept of flicker 50% of the time could be very detrimental for the observer. Aiming for a lower visibility rate of flicker would mean threshold estimates of higher frequency than those presented thus far. Since in our experiments we measured the full psychometric function using the constant stimuli method, it is possible to offer estimates of the frequencies of flicker that would lead to only a 10% visibility rate of flicker (i.e., the lowest frequency at which participants report to not see flicker in 90% of the trials). This percentage was selected as it is an alternative threshold occasionally reported in the literature [8].

**Figure 6:**
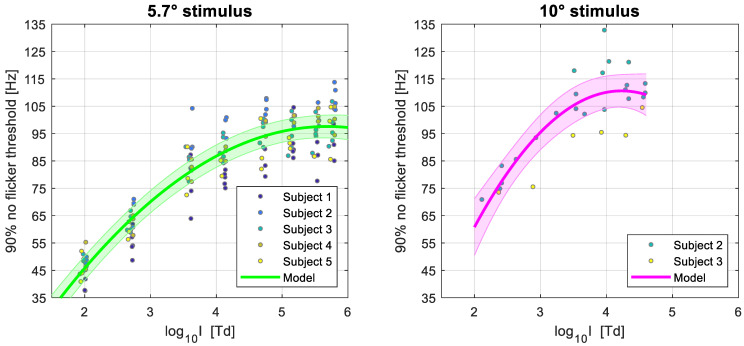
Quadratic linear mixed model results of the 90% “no flicker” threshold as a function of log_10_ retinal illuminance, for stimulus sizes of 5.7 (**left**) and 10 (**right**) degrees of visual angle. The continuous colored lines represent the model fit and the shaded regions represent the 95% confidence intervals. The individual markers show the estimated thresholds for each subject over several experimental sessions, and the color of the markers represents the participant as indicated by the legend.

In Figure 6, we illustrate these estimates for the 5.7° and 10° stimuli, as well as the linear mixed models fitted with the same parameters as the previous ones presented in this section. The estimates and full results of the model are shown in Table 3.

As expected, we observe higher values of frequencies that can be perceived when taking the lower visibility rate of 10%, albeit the effect is modest, with the frequency at which the models saturate increasing by ~10 Hz in both target sizes. From the estimated parameters one can estimate the minimum frequency at which flicker is perceived <10% of the time for different values of retinal illuminance and for an average observer given our sample. 

## 4. Discussion

The relationship between light intensity and the temporal sensitivity of the human visual system has very important practical applications, particularly in the design of digital displays and illumination sources that are temporally modulated. This relationship is best described by the Ferry–Porter law, which states that the CFF increases linearly with the logarithm of the retinal illuminance. This linearity has been shown to hold for a wide range of stimuli and up to log_10_ 4 Trolands [2]; however, beyond this intensity, it is unknown if the CFF continues to rise linearly or if saturation is reached. In this study, we aimed to extend the experimental data available on the peripheral CFF at higher light intensity levels than previously reported in the literature. For this, we built an experimental setup following the one described by Tyler et al. [2], with careful control of the illumination source, the stimulus size and location, and the psychophysical method used to estimate the CFF [18]. 

To analyse our results, we first fitted a piece-wise linear regression with two segments. For the slope of the first segment, we found results comparable to those of Tyler et al. [2], with the CFF increasing by 19 to 25 Hz/decade with increasing retinal illuminance for both target sizes. Their sample showed rates of increase of ~20 Hz/decade for the same temporal retinal eccentricity of 35°. However, while these authors show that the linear increase continues for up to log_10_ 4 Trolands, we found that the saturation of the response can start at lower illuminances for some subjects. The estimated breakpoint or sudden change in the rate of the response was between log_10_ 3.6 and 4.4 Trolands among our sample. Beyond this, the response saturates and the rate of increase in the CFF decreases dramatically.

The change in the rate of the response was in reality not a sudden transition, but rather a gradual change. To capture this, we fitted a linear mixed model with a quadratic term. We found that saturation happened gradually between ~log_10_ 3.6 and log_10_ 4.6 Trolands, with the CFF reaching just below 90 Hz for the target of 5.7 degrees of visual angle, and approximately 100 Hz for the 10 degrees target. As expected, the thresholds were higher for the test field with larger area, but the speed of the response was the same, which was reflected in higher absolute values of CFF but similar slope estimates. This is anticipated to happen for a stimulus of a larger area but equal retinal eccentricity and wavelength of light. In practical terms, as visual displays tend to have larger sizes than the stimuli used, we can expect the maximum frequency at which saturation occurs to be higher.

Another important practical consideration is that the way thresholds are usually defined might not be optimal for the design of visual displays. A correct response rate of 75% is a common specification for psychophysical thresholds. In a YES/NO task, this corresponds to a 50% of flicker visibility; that is, subjects report perceiving flicker half of the time. Taking this into account, and as we measured the full psychometric function, we also reported the frequency at which flicker was only visible at a rate of 10%, which increased the estimated values by approximately 10 Hz.

Finally, it is important to note that while the estimates offered refer to an average observer given our sample, in practical applications more weight might be given to the more sensitive observers; as for the design of visual displays, one would want to avoid the visibility of artifacts in the large majority of users. For this purpose, the estimates obtained through the linear mixed model for individual participants might be a useful contribution.

As modern digital displays continue to increase in brightness, it is important to know how this can affect the visibility of flicker and other temporal artifacts. While models exist that can predict how the CFF will change depending on target size and eccentricity [7,8], they also assume that the Ferry–Porter law will continue to hold with increasing log illuminance. However, we find here that the response in fact saturates, decreasing the need for proportional increases in the refresh rates of displays, albeit these would still need to higher than the CFF in order to avoid other temporal artifacts in the image.

## Figures and Tables

**Table 1:** Segmented linear regression results of CFF as a function of log10 retinal illuminance for each size of stimuli and subject. The breakpoint is the value of log I at which the piece-wise linear function separates. The estimated values (Est.), their standard errors (SE), and 95% confidence intervals (CI 95%) are shown. The slopes are in units of Hz/decade, and the x-intercept and breakpoint in units of log10 Trolands.

Target Size/ Subject		Breakpoint [log_10_ Td]				Segment 1						Segment 2	
						Slope [Hz/decade]				x-Intercept [log_10_ Td]		Slope [Hz/dec.]	
		Est.	95% CI		SE	Est.	95% CI		SE	Est.	SE	Est.	SE
**5.7°**	1	3.61	2.85	4.37	0.4	21.4	12.9	29.8	4.2	0.36	0.47	6.3	1.1
	2	3.71	3.55	3.86	0.1	25.5	23.5	27.5	1.0	0.42	0.11	3.3	0.8
	3	3.73	3.51	3.96	0.1	20.7	18.6	22.7	1.0	−0.01	0.14	4.9	0.9
	4	4.45	4.22	4.68	0.1	18.2	16.6	19.8	0.8	−0.19	0.13	1.1	1.6
	5	3.63	3.15	4.11	0.2	19.2	15.3	23.2	1.9	−0.13	0.28	5.1	1.6
	Mean	3.82	3.45	4.20	0.2	21.0	17.4	24.6	1.8	0.09	0.23	4.2	1.2
**10°**	2	3.87	3.67	4.06	0.1	24.0	19.5	28.4	2.1	−0.54	0.26	−9.2	4.5
	3	3.73	3.27	4.20	0.1	19.5	15.1	23.9	1.0	−0.82	0.15	6.2	1.8
	Mean	3.80	3.47	4.13	0.1	21.7	17.3	26.1	1.6	−0.68	0.21	−1.5	3.2

**Table 2:** Linear mixed model results of CFF as a function of log10 retinal illuminance and squared log10 retinal illuminance. The parameters estimate (Est.), their standard errors (SE) and 95% confidence intervals (95% CI) are shown, as well as the t-test results and the standard deviation of the different random effects included (RE SD).

5.7° Stimulus—CFF [Hz]									
Parameter	Est.	SE	95% CI		*t*-Ratio	df	*p*-Value	RE SD	
								Subject	Session
Intercept	−26.05	3.42	−32.80	−19.30	−7.63	157	<0.001	3.03	0.86
log_10_ I	39.86	1.71	36.49	43.24	23.34	157	<0.001	-	-
(log_10_ I)^2^	−3.57	0.22	−3.99	−3.14	−16.58	157	<0.001	-	-
**10° Stimulus—CFF [Hz]**									
**Parameter**	**Est.**	**SE**	**95% CI**		***t*** **-Ratio**	**df**	***p*** **-Value**	**RE SD**	
								**Session**	
Intercept	−70.52	14.03	−99.26	−41.77	−5.03	28	<0.001	4.05	
log_10_ I	80.80	8.38	63.64	97.97	9.64	28	<0.001	-	
(log_10_ I)^2^	−9.59	1.21	−12.07	−7.11	−7.92	28	<0.001	-	

**Table 3:** Linear mixed model results of the 90% “no flicker” threshold as a function of log10 retinal illuminance and squared log10 retinal illuminance. The parameters estimate (Est.), their standard errors (SE) and 95% confidence intervals (95% CI) are shown, as well as the t-test results and the standard deviation of the different random effects included (RE SD).

5.7° Stimulus—90% “No Flicker” Threshold [Hz]									
Parameter	Est.	SE	95% CI		*t*-Ratio	df	*p*-Value	RE SD	
								Subject	Session
Intercept	−25.64	5.19	−35.89	−15.39	−4.94	153	<0.001	4.28	1.25
log_10_ I	43.34	2.64	38.13	48.55	16.44	153	<0.001	-	-
(log_10_ I)^2^	−3.81	0.33	−4.47	−3.15	−11.46	153	<0.001	-	-
**10° stimulus—90% “no flicker” threshold [Hz]**									
**Parameter**	**Est.**	**SE**	**95% CI**		***t*** **-Ratio**	**df**	***p*** **-Value**	**RE SD**	
								**Session**	
Intercept	−68.72	28.14	−126.67	−10.77	−2.44	25	0.022	5.37	
log_10_ I	84.82	17.07	49.66	119.98	4.97	25	<0.001	-	
(log_10_ I)^2^	−10.03	2.49	−15.16	−4.90	−4.03	25	<0.001	-	

## Data Availability

The data presented in this research article and the processed results are available in the Newcastle University research repository with the identifier https://doi.org/10.25405/data.ncl.21725336 [23]. The code used for the analyses and to generate the figures are also available in this repository with the identifier https://doi.org/10.25405/data.ncl.21764885 [24].
